# Enhanced Immunomodulatory Effect of Intravenous Immunoglobulin by Fc Galactosylation and Nonfucosylation

**DOI:** 10.3389/fimmu.2022.818382

**Published:** 2022-01-28

**Authors:** Yusuke Mimura, Yuka Mimura-Kimura, Radka Saldova, Pauline M. Rudd, Roy Jefferis

**Affiliations:** ^1^ Department of Clinical Research, National Hospital Organization Yamaguchi Ube Medical Center, Ube, Japan; ^2^ NIBRT GlycoScience Group, National Institute for Bioprocessing Research and Training, Dublin, Ireland; ^3^ UCD School of Medicine, College of Health and Agricultural Science, University College Dublin, Dublin, Ireland; ^4^ Bioprocessing Technology Institute, Agency for Science, Technology and Research, Centros, Singapore; ^5^ Institute of Immunology and Immunotherapy, College of Medical and Dental Sciences, University of Birmingham, Birmingham, United Kingdom

**Keywords:** glycoengineering, antibody-dependent cellular cytotoxicity, intravenous immunoglobulin, autoimmune disease, natural killer cell, Fcγ receptor, oligosaccharide

## Abstract

Intravenous immunoglobulin (IVIG) is used as an immunomodulatory agent in the treatment of various autoimmune/inflammatory diseases although its mechanism of action remains elusive. Recently, nonfucosylated IgG has been shown to be preferentially bound to Fcγ receptor IIIa (FcγRIIIa) on circulating natural killer cells; therefore, we hypothesized that nonfucosylated IVIG may modulate immune responses through FcγRIIIa blockade. Here, homogeneous fucosylated or nonfucosylated glycoforms of normal polyclonal IgG bearing sialylated, galactosylated or nongalactosylated Fc oligosaccharides were generated by chemoenzymatic glycoengineering to investigate whether the IgG glycoforms can inhibit antibody-dependent cellular cytotoxicity (ADCC). Among the six IgG glycoforms, galactosylated, nonfucosylated IgG [(G2)_2_] had the highest affinity to FcγRIIIa and 20 times higher potency to inhibit ADCC than native IgG. A pilot study of IVIG treatment in mice with collagen antibody-induced arthritis highlighted the low-dose (G2)_2_ glycoform of IVIG (0.1 g/kg) as an effective immunomodulatory agent as the 10-fold higher dose of native IVIG. These preliminary results suggest that the anti-inflammatory activity of IVIG is in part mediated *via* activating FcγR blockade by galactosylated, nonfucosylated IgG and that such nonfucosylated IgG glycoforms bound to FcγRs on immune cells play immunomodulatory roles in health and disease. This study provides insights into improved therapeutic strategies for autoimmune/inflammatory diseases using glycoengineered IVIG and recombinant Fc.

## Introduction

IVIG is a therapeutic preparation of normal polyclonal IgG derived from pooled plasma of thousands of healthy donors and is administered at a high dose for the treatment of autoimmune/inflammatory disorders, including immune thrombocytopenia (ITP), Kawasaki Disease and Guillain-Barré syndrome (1–4). The anti-inflammatory activity of IVIG is shown to reside in the Fc portion of IgG from a clinical study on the treatment of ITP with the Fc fragments (5). Although various mechanisms of action of IVIG have been proposed, including blockade of activating FcγRs (6–8), expansion of regulatory T cells (9–11), and upregulation of inhibitory FcγRIIb *via* sialylated IgG binding to type II lectin receptors (12, 13), the precise mechanism of action of IVIG in autoimmune diseases remains inconclusive (2, 3, 14).

A possible differential role has been proposed for Fc oligosaccharides of IgG to influence the immunomodulatory effect of IVIG (3, 15, 16). The oligosaccharide attached at Asn297 residue of each C_H_2 domain of IgG-Fc is essential for optimal expression of biological activities mediated through FcγRs (FcγRI, FcγRIIa/b/c, FcγRIIIa/b) and the C1q component of complement (17–20). The Fc oligosaccharides of serum-derived IgG are highly heterogeneous due to variable addition and processing of outer-arm sugar residues [sialic acid, galactose and bisecting N-acetylglucosamine (GlcNAc)] and fucose onto the core diantennary heptasaccharide (GlcNAc_2_Mannose_3_GlcNAc_2_, designated G0) (**Supplementary Figure 1** and **Supplementary Table 1**) (21). The differentially glycosylated species (glycoforms) of IgG-Fc express unique biological activities, modulating antibody effector functions including ADCC and complement-dependent cytotoxicity (17, 18, 20, 22). In particular, nonfucosylation of IgG-Fc increases FcγRIIIa binding and ADCC ~50-fold (23, 24), which has been exploited for the development of therapeutic recombinant monoclonal antibodies for treatment of cancers, inflammatory and infectious diseases (25–28). On the other hand, biological significance of naturally occurring nonfucosylated glycoforms present at 5 – 10% of serum IgG (or IVIG) remains unclear. Recently, the majority of IgG antibodies bound to FcγRIIIa on circulating natural killer cells have been shown to be nonfucosylated, in contrast to those in the sera of the same subjects which are mostly fucosylated (29). Here, we hypothesized that nonfucosylated IgG in serum can saturate FcγRIIIa on immune cells due to its high affinity and modulate immune responses. We demonstrate that nonfucosylated glycoforms of normal polyclonal IgG can markedly inhibit ADCC compared with the fucosylated glycoforms. Notably, the galactosylated, nonfucosylated (G2)_2_ glycoform exhibits a significant therapeutic efficacy *in vivo* at a low dose and is comparable to the 10-fold higher dose of native IVIG. These results provide improved therapeutic strategies for autoimmune diseases using IVIG. The anti-inflammatory activity of the (G2)_2_ glycoform sheds light on the association between glycosylation changes of total serum IgG and the pathophysiology of certain autoimmune diseases.

## Methods

### Expression of EndoS, EndoS D233Q and α-L-Fucosidase AlfC

Expression vectors pET-30a(+)-ndoS D233Q and pET28a(+)-α-L-fucosidase encoding EndoS D233Q from *Streptococcus pyogenes* and α-L-fucosidase AlfC from *Lactobacillus casei*, respectively, were generously provided by Dr. Wei Huang (30, 31). Expression vector encoding EndoS wildtype was prepared by site-directed mutagenesis using pET-30a(+)-ndoS D233Q, Quickchange Lightning site-directed mutagenesis kit (Agilent), forward primer 5′-GGCCTGGACGTTGACGTGGAACACGATAGCATTCCGAAAGTG-3′, and reverse primer 5′-TTCCACGTCAACGTCCAGGCCATCCAGGTTGTACTTGTACAC-3′. The vectors were transformed into BL21(DE3) competent cells (Novagen), and the enzymes were expressed and purified as previously described (30, 31).

### Preparation of Glycan Oxazolines

The glycan donors sialoglycan oxazoline (S2G2-Ox), galactosylated glycan oxazoline (G2-Ox), and nongalactosylated glycan oxazoline (G0-Ox) were prepared from sialylglycopeptide (SGP) (Tokyo Chemical Industry Co. Ltd.) in a modified version of the previously described method (32). Briefly, SGP (20 mg) dissolved in 100 μl of 50 mM phosphate (pH 6.0) was digested at 37°C for 8 h with EndoS-coupled Sepharose-4 that had been prepared by coupling EndoS to CNBr-activated Sepharose-4 (GE Healthcare) to release sialoglycan, according to the manufacturer’s instruction. For G2-Ox and G0-Ox preparation, SGP (40 mg) was digested with EndoS-coupled Sepharose-4 and neuraminidase (2 U, Roche) overnight and the supernatant containing the desialylated glycan was divided into two aliquots, with one for preparation of G2-Ox and the other for G0-Ox. For the latter, the galactosylated glycan was digested with β (1-3,4)-galactosidase (Agilent) at 37°C for 48 h. The glycan in each aliquot (~100 µl) was converted to glycan oxazoline by the addition of 2-chloro-1,3-dimethylimidazolinium chloride (23.4 mg) and triethylamine (47.2 μl) on ice for 1 h. The reaction was diluted with 4 ml of butanol:ethanol:water (4:1:1, v/v/v) and purified on cellulose column (2 ml in a Poly-Prep Chromatography Column, Bio-Rad) equilibrated with the same solution (33). After washing the column with 12 ml of the solution and 2 ml of absolute ethanol, glycan oxazoline was eluted with distilled water. The glycan-containing fractions were detected with anthrone/sulfuric acid and dried under vacuum.

### Preparation of Homogeneous Glycoforms of Normal IgG

A series of fully sialylated and the truncated glycoforms of normal IgG were prepared by chemoenzymatic glycoengineering, according to the previously described method (30). Briefly, commercial IVIG (Gammagard, Shire Japan) dissolved at ~40 mg/ml in 50 mM acetate, 5 mM CaCl_2_ (pH 5.5) was deglycosylated with EndoS-coupled Sepharose-4 at 37°C for 8 h to prepare IgG bearing Fuc-GlcNAc at Asn297 (Fuc-GlcNAc-IgG), then dialyzed against 50 mM Tris-HCl (pH 7.4). To prepare IgG bearing GlcNAc (GlcNAc-IgG) it was further digested with α-L-fucosidase AlfC at 37°C for 48 h. For transglycosylation, either GlcNAc-IgG or Fuc-GlcNAc-IgG at ~25 mg/ml was incubated with 0.6 mg/ml EndoS D233Q in the presence of 3 mM glycan oxazoline at 30°C for 4 h. The completion of transglycosylation was confirmed by SDS-PAGE and the remodeled IgG glycoforms were purified on protein G-Sepharose 4 Fast flow column (GE Healthcare).

### Glycan Analysis of Homogeneous IgG Glycoforms

IgG (1 mg) was digested with papain (20 μg) in 0.1 M phosphate, 0.15 M NaCl, 2 mM EDTA (pH 7.0) at 37°C overnight, then treated with 50 μM iodoacetamide for 30 min on ice, and dialyzed against 10 mM phosphate buffer (pH 8.0). The Fab and Fc were separated by diethylaminoethyl-cellulose anion exchange chromatography (DE52; Whatman Biosystems, Chalfont St Giles, UK) equilibrated with the same buffer. The dialyzed papain digest was applied to the column, and the Fab was obtained in the fall-through fractions. After washing the column with five column volumes of 10 mM phosphate (pH 8.0), 10 mM phosphate-buffered saline (pH 7.4) (PBS) was added to elute the Fc (21). The oligosaccharides were released with peptide-*N*-glycosidase F from the Fc of an individual IgG glycoform in the SDS-PAGE gel bands and labeled with 2-aminobenzamide (2-AB) by using Signal 2-AB plus labeling kit (Agilent) as previously described (34). The fluorescently labeled oligosaccharides were separated by using a Waters ACQUITY H-class Bio ultraperformance liquid chromatography (UPLC) system on a sub-2 μm hydrophilic interaction based stationary phase with a Waters ACQUITY UPLC Glycan BEH Amide column (2.1 × 150 mm i.d., 1.7 μm BEH particles) as previously described (35). The oligosaccharide peaks were assigned in accordance with the previous study (36).

### Fcγ Receptor (FcγR) Binding Assays

The binding of the IgG glycoforms to FcγRs was analyzed as previously described (37). Briefly, recombinant human FcγR proteins (FcγRIIIa V158/F158 and FcγRIIa R131/H131) (R&D Systems) at 2.5 – 5 μg/ml in PBS were coated on high-binding microtiter plates (Corning 3690 High Binding Half Area) overnight at 4°C. The FcγR-coated plates were washed with PBS containing 0.05% Tween 20 (PBS-T) three times and blocked with PBS containing 1% bovine serum albumin for 1 h at room temperature. Serially diluted IgG glycoforms were added to the FcγRIIIa-coated plates and allowed to bind for 2 h at 37°C. After washing with PBS-T three times, the bound IgG was detected with goat F(ab’)_2_ anti-human IgG F(ab’)_2_-peroxidase conjugate (Abcam). After incubation for 2 h at 37°C, the plates were washed five times with PBS-T and developed with 50 μl of 3,3′,5,5′-tetramethylbenzidine substrate per well, which was stopped by the addition of 12.5 μl of 12.5% H_2_SO_4_ per well. Absorbance was measured at 450 nm on a Multiskan™ microplate reader (Thermo Fisher Scientific). The concentration of IgG corresponding to half-maximal binding on the ELISA binding curve was considered as an apparent affinity to the respective FcγR and was compared between the IgG glycoforms.

### ADCC Reporter Bioassay

ADCC reporter bioassay mediated by FcγRIIIa V158 or F158 was performed, according to the manufacturer’s instruction (Promega). Briefly, CD20-expressing Raji cells grown in RPMI1640 cell culture medium supplemented with 10% heat-inactivated fetal bovine serum (Gibco), 2 mM glutamine, 100 μg/ml penicillin and 100 U/ml streptomycin (10% RPMI) were plated after washing once with PBS and resuspended in RPMI1640 medium containing 4% fetal bovine serum, ultra-low IgG (Life Technologies) at 12,500 cells/25 μl/well in white opaque tissue culture plates (BD Falcon 353296), followed by the addition of 25 μl of rituximab (anti-CD20 IgG) that was 4-fold serially diluted from the starting concentration of 10 μg/ml with the same medium. An individual normal IgG glycoform dissolved in PBS was added to each well (7.5 μl/well). Jurkat cells stably expressing human FcγRIIIa V158 (or F158) and NFAT-luciferase reporter in 10% RPMI were added at 75,000 cells/17.5 μl/well to rituximab-opsonized Raji cells at 37°C, 5% humidified CO_2_ for 6 h. BioGlo luciferase assay reagent was added (75 μl/well), and chemiluminescence was measured with a luminometer (Fluoroskan Ascent FL, Thermo Fisher Scientific). Inhibition of ADCC was examined with increasing concentrations of native IgG (0 – 10 mg/ml), with various fucosylation levels of sialylated IgG or galactosylated IgG (0%, 25%, 50%, and 100%) at 0.2 mg/ml, and with the six individual IgG glycoforms at 0.1 mg/ml. Additionally, titration of the IgG glycoforms (0 – 2 mg/ml) was performed to compare the ADCC inhibitory capability at 0.1 μg/ml rituximab.

### Statistical Analysis

The ELISA data for the IgG glycoforms–FcγR interactions and the ADCC reporter bioassay data were fitted to sigmoidal dose-response curves (GraphPad Prism v6). The differences in the concentration of rituximab that gave 50% of the maximal response (EC_50_) in the presence or absence of the glycoforms of IgG were tested by the extra sum of squares *F*-test (GraphPad Prism v6). Likewise, the differences in 50% inhibitory concentration (IC_50_) of the IgG glycoforms for inhibition of the ADCC reporter activity were tested. *p*<0.05 was considered statistically significant.

## Results

### Remodeling of IgG Glycosylation by Chemoenzymatic Glycoengineering

**Figure 1:**
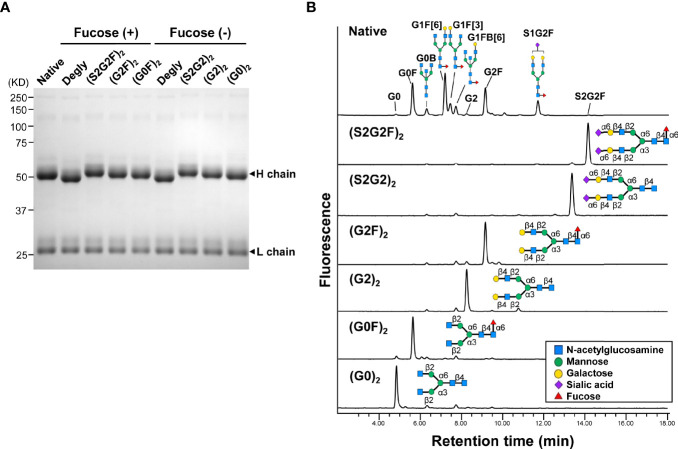
Homogeneous glycoforms of normal polyclonal IgG prepared by chemoenzymatic glycoengineering. **(A)** SDS-PAGE of the glycoforms of IgG. All the IgG glycoforms including the native protein used in this study were purified by protein G affinity chromatography. **(B)** HILIC-UPLC analysis of glycans released from the Fc fragments of IgG glycoforms. The peaks of the oligosaccharides of native IgG are listed in **Supplementary Table 1**.

A glycoform of normal polyclonal IgG bearing homogeneous oligosaccharide chains (S2G2)_2_, (S2G2F)_2_, (G2)_2_, (G2F)_2_, (G0)_2_, or (G0F)_2_ was prepared by transfer of the glycan donor S2G2-Ox, G2-Ox or G0-Ox to fucosylated or nonfucosylated GlcNAc residues of IgG with EndoS D233Q. Complete transfer of the respective glycans was confirmed by SDS-PAGE (**Figure 1A**) and the structures of the glycans released with peptide-*N*-glycosidase F from the Fc fragments were analyzed by HILIC-UPLC, exhibiting a single peak of each glycoform, in contrast to heterogeneous peaks of native IgG (**Figure 1B**, **Supplementary Figure 1**, and **Supplementary Table 1**).

### Binding of IgG Glycoforms to Human FcγRs

**Figure 2:**
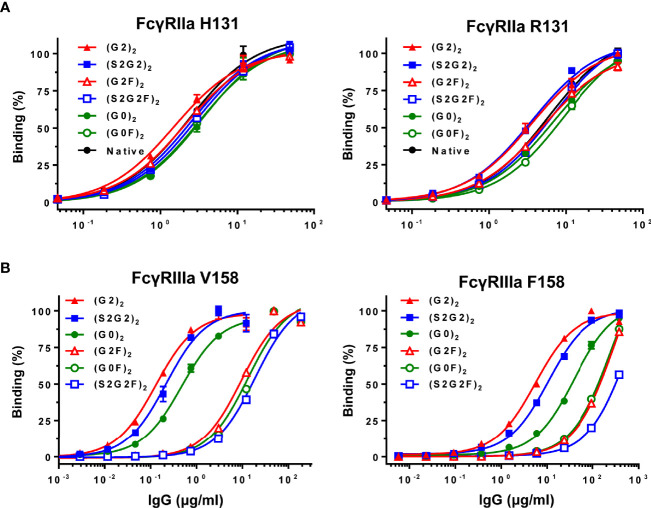
Binding of IgG glycoforms to FcγRs. **(A)** FcγRIIa H131 and R131 variants. **(B)** FcγRIIIa V158 and F158 variants. All data points represent the calculated mean of two independent measurements from a total of at least two experiments. The data were fitted to a sigmoidal dose-response curve (GraphPad Prism).

Binding to FcγRIIa (H131 or R131) or FcγRIIIa (V158 or F158) was compared between the six IgG glycoforms and native IgG by ELISA (**Figure 2**). All the IgG glycoforms exhibited comparable FcγRIIa binding profiles to native IgG, which confirms no adverse effect of the glycoengineering processes on the FcγR binding capability of the remodeled IgG glycoforms (**Figure 2A**). Galactosylation had positive influence on FcγRIIa binding (**Figure 2A**) while the nongalactosylated glycoforms [(G0)_2_ and (G0F)_2_] had generally lower affinity, with the differences in the apparent affinity between the (G2)_2_ and the (G0F)_2_ being ~2-fold for both FcγRIIa H131 and R131 variants. On the other hand, nonfucosylation had profound influence on FcγRIIIa binding, with the differences in the apparent affinity between the nonfucosylated glycoforms and the fucosylated counterparts being 30 – 70-fold for the V158 variant and 4 – 30-fold for the F158 variant (**Figure 2B**). Notably, the (G2)_2_ glycoform had the highest affinity to both FcγRIIIa V158 and F158 variants while the sialylated, fucosylated (S2G2F)_2_ glycoform had the lowest affinity to FcγRIIIa (**Figure 2B**).

### The (G2)_2_ Glycoform of Normal IgG Potently Inhibits ADCC

**Figure 3:**
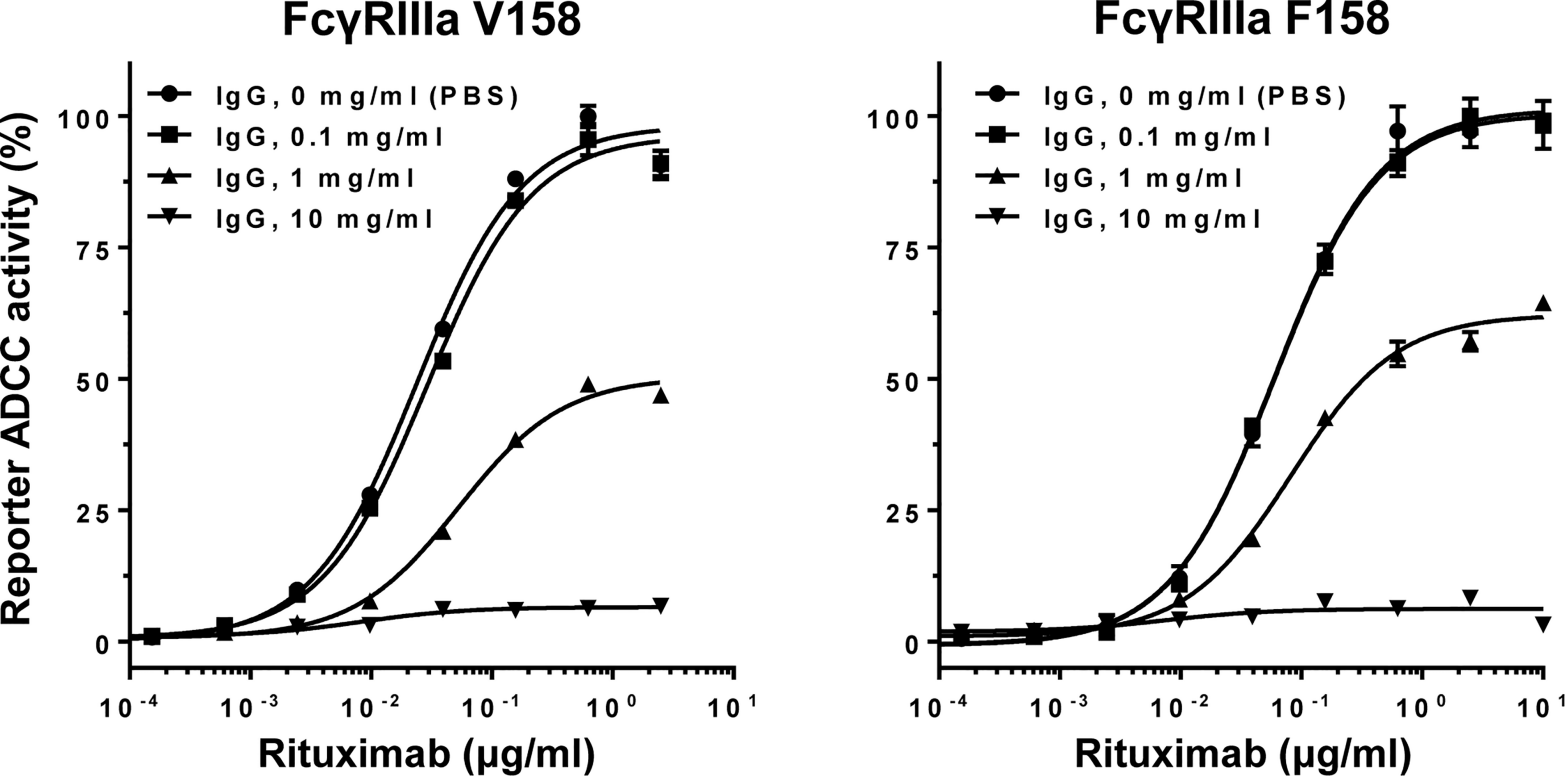
Normal polyclonal IgG inhibits ADCC in a dose-dependent manner. Note that activation of FcγRIIIa V158 or F158 variant was inhibited by normal IgG at >0.1 mg/ml. Error bars, mean ± S.E. (n = 3).

The influence of normal polyclonal IgG on ADCC was examined with increasing concentrations of normal IgG in rituximab (anti-CD20 antibody)-mediated, FcγRIIIa-based ADCC reporter bioassay. Inhibition of ADCC was observed in a dose-dependent manner for both FcγRIIIa V158 and F158 variants where the EC_50_ values progressively increased in a range of 0.1–1 mg/ml of normal IgG (**Figure 3**).

**Figure 4:**
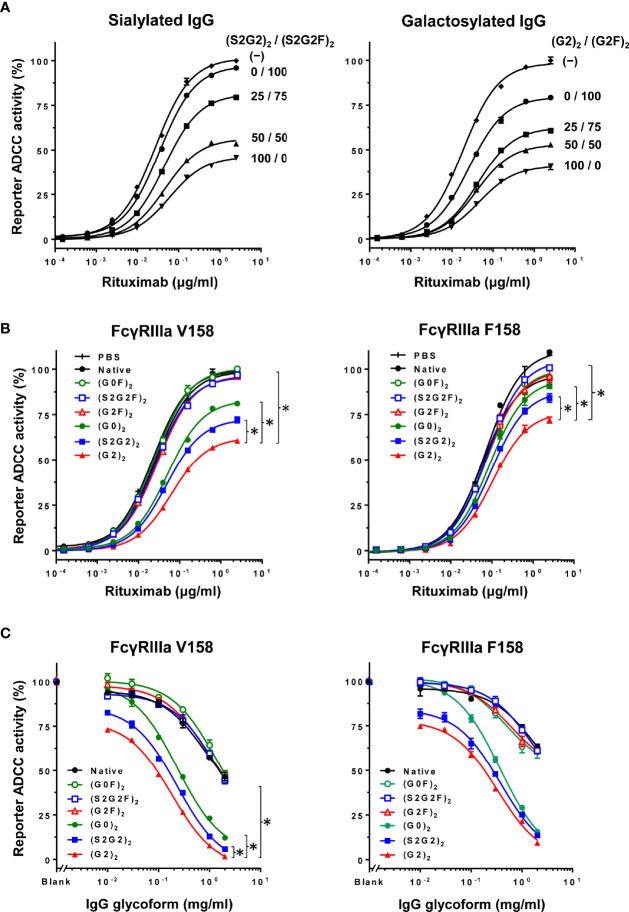
Inhibition of ADCC with normal IgG is glycoform-dependent. **(A)** Influence of fucosylation of normal IgG on inhibition of ADCC was examined by titration of fucosylation levels of sialylated glycoforms (left) and galactosylated glycoforms (right) at the final concentration of 0.2 mg/ml. Error bars, mean ± S.E. (n = 3). **(B)** Influence of the IgG glycoforms on inhibition of ADCC was examined at 0.1 mg/ml of each IgG glycoform. Error bars, mean ± S.E. (n = 3). Note that the differences in EC_50_ between the (G2)_2_ and other glycoforms were significant (asterisks) for both FcγRIIIa V158 and F158 (p < 0.01) as determined by extra sum of squares *F*-test. **(C)** Titration of the IgG glycoforms for comparison of the ADCC inhibitory capability at 0.1 μg/ml rituximab. Error bars, mean ± S.E. (n = 3). *p < 0.05.

The influence of fucosylation of normal IgG on ADCC inhibition was examined by titration of the fucosylation levels of sialylated or galactosylated IgG at 0.2 mg/ml (**Figure 4A**). Decrease in the fucosylation levels resulted in progressive increases in the inhibitory activity for both sialylated and galactosylated IgG. This result clearly indicates that the glycoform of normal IgG is important for modulation of ADCC.

Inhibition of ADCC was further examined using the six IgG glycoforms at 0.1 mg/ml (**Figure 4B**). ADCC was markedly inhibited with non-fucosylated IgG [(S2G2)_2_, (G2)_2_ and (G0)_2_] as compared with the fucosylated IgG counterparts [(S2G2F)_2_, (G2F)_2_ and (G0F)_2_]. Additionally, titration of these IgG glycoforms was performed to compare the IC_50_ for ADCC inhibition between the IgG glycoforms (**Figure 4C**). The IC_50_ values obtained for (G2)_2_, (S2G2)_2_, (G0)_2,_ and native IgG were 0.1, 0.16, 0.28 and 2.0 mg/ml, respectively. This indicates that the inhibitory capacities of the (G2)_2_, (S2G2)_2_, and (G0)_2_ glycoforms are 20, 12.5, and 7-fold higher than that of native IgG, respectively. Notably, galactosylation and nonfucosylation of normal IgG resulted in the most potent inhibition of ADCC (**Figures 4B, C**), which is explained by its enhanced affinity for FcγRIIIa (**Figure 2B**). In contrast, sialylation or nongalactosylation of IgG had a subtle but negative impact on the inhibition of ADCC (**Figures 4B, C**), which corresponds to the decreased affinities to FcγRIIIa (**Figure 2B**).

On the other hand, FcγRIIa-mediated antibody-dependent cellular phagocytosis (ADCP) was inhibited by normal IgG at >1 mg/ml (**Supplementary Figure 2A**); however, ADCP was not modulated by IgG glycoforms (**Supplementary Figure 2B**).

### (G2)_2_ IVIG Attenuates Collagen Antibody-Induced Arthritis in Mice

Whether the IgG glycoforms exert anti-inflammatory effects was examined in mice with collagen antibody-induced arthritis (CAIA) (**Supplementary Figure 3**). Low-dose (0.1 g/kg) IgG glycoforms [(G2)_2_, (S2G2)_2_, (S2G2F)_2_, native] and high-dose (1 g/kg) native IgG as positive control were administered to the mice, and the group receiving the (G2)_2_ glycoform had the lowest arthritis score and serum interleukin-6 levels among the groups (**Supplementary Figure 3**).

## Discussion

**Figure 5:**
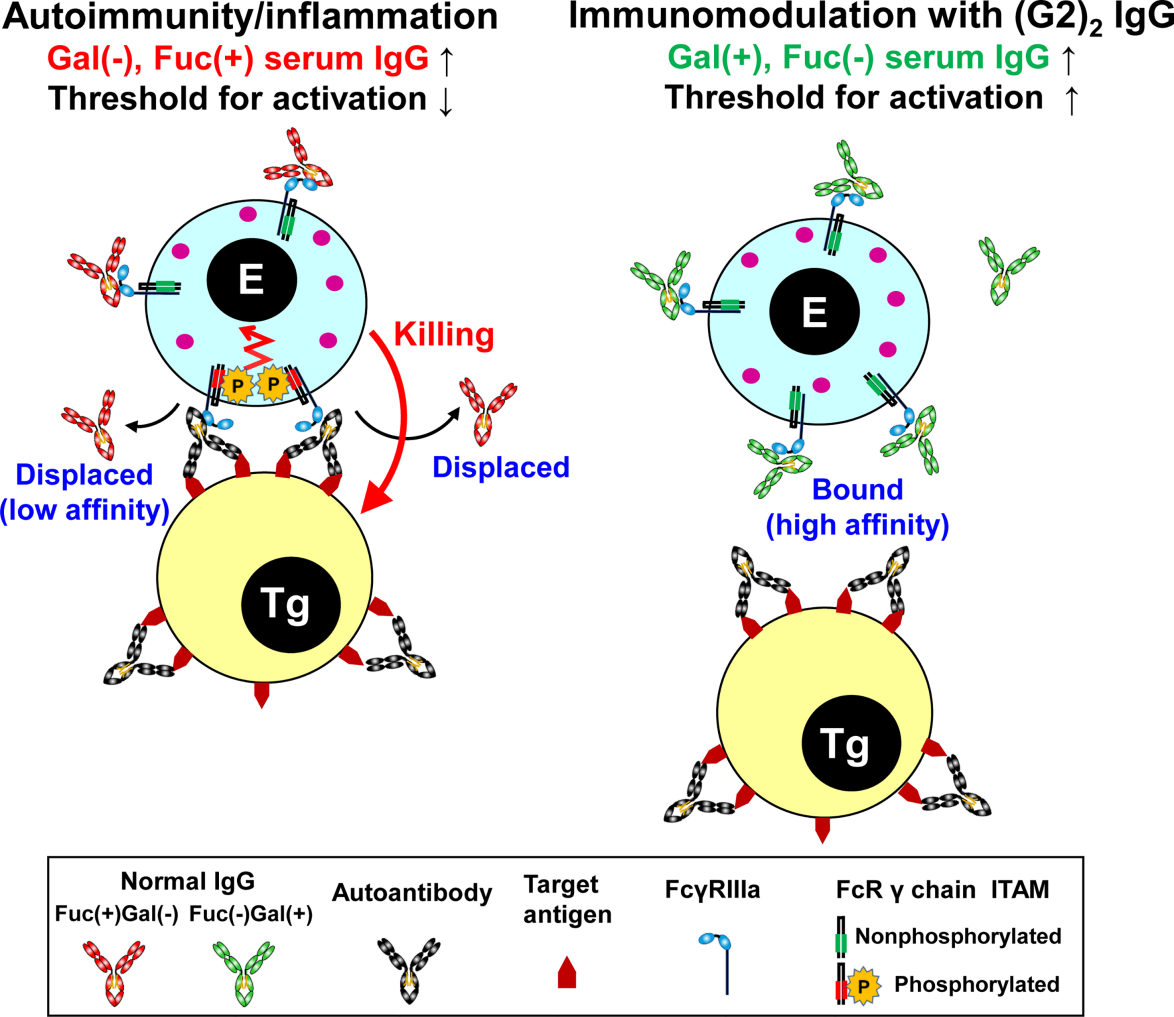
Summary of the mechanism of immunomodulation by the (G2)_2_ glycoform of IgG. In autoimmune/inflammatory state, hypogalactosylated, fucosylated serum IgG cannot compete with high-avidity multimeric IgG immune complexes on a target cell for FcγRIIIa binding, resulting in the activation of an effector cell (left). Increased levels of galactosylated, nonfucosylated serum IgG by administration of the (G2)_2_ glycoform of IVIG result in saturation of FcγRIIIa with the (G2)_2_ glycoform, inhibiting the activation of an effector cell (right). E, effector cell. Tg, target cell. Gal, galactose. Fuc, fucose.

A rationale for the use of IVIG, at a high dose, and its mechanism of action in the treatment of autoimmune/inflammatory diseases remain to be elucidated. We have shown robust immunomodulatory activity of the galactosylated, non-fucosylated (G2)_2_ glycoform of human normal IgG as a minor but active component of IVIG. High affinity-binding of galactosylated, nonfucosylated IgG to FcγRIIIa that can modulate immune responses including ADCC is a novel mechanism of action of IVIG (**Figure 5**). This study provides insights into improved therapeutic strategies for autoimmune diseases and the involvement of endogenous galactosylated, nonfucosylated IgG in immune homeostasis.

The immunomodulatory effect of IVIG was Fc glycoform-dependent. The (G2)_2_ glycoform of IVIG at a low dose (0.1 g/kg) was as protective as the 10-fold higher dose of native IVIG in mice with collagen antibody-induced arthritis (**Supplementary Figure 3**). The robust anti-inflammatory activity of the (G2)_2_ glycoform is consistent with the highest affinity to FcγRIIIa (38–40) and the strongest ADCC inhibitory activity among the six IgG glycoforms examined (**Figures 2B**, **4B, C**). The mice in the (S2G2)_2_ and (S2G2F)_2_-treated groups were not protected (**Supplementary Figure 3**), which is consistent with previous reports that the anti-inflammatory activity of IVIG is independent of Fc sialylation (41–43) but not with the report by Washburn et al. about enhanced anti-inflammatory effects of hyper-sialylated Fc (44). However, the difference in the outcome between these studies might be attributed to different sialylated IgG/Fc preparations and experimental protocols.

Galactosylation and nonfucosylation influence FcγRIIIa binding independently because the α(1-6)-arm galactose interacts with the amino acid residues at the C_H_2/C_H_3 domain interface while core fucose is proximal to the lower hinge region. Lack of core fucose of IgG-Fc increases oligosaccharide–oligosaccharide and oligosaccharide–protein interactions between FcγRIIIa and IgG-Fc, thereby stabilizing complex formation (45, 46). On the other hand, the galactose residue(s) contribute to the stability of IgG-Fc structure, as evidenced by increased enthalpy for the unfolding of the galactosylated C_H_2 domains (40, 47), increased mobility of the Fc oligosaccharide by removal of galactose (48), and lowered deuterium uptake in the hydrophobic surface of the galactosylated C_H_2 domain spanning Phe241 to Met252 (49). By crystallographic analysis, the α(1-6)-arm galactose makes 27 non-covalent contacts with the protein structure of the C_H_2 domain including a minimum of 2 hydrogen bonds (50). Additionally, the two C_H_2 domains of the (G2F)_2_ glycoform adopts an open conformation of the horseshoe-shaped Fc, which is favorable for FcγRIII binding (51, 52). In contrast, sialylation of the Fc had a minor but negative impact on FcγRIIIa binding, resulting in lowered ADCC inhibitory activity as compared with the (G2)_2_ glycoform (**Figures 2B**, **4B, C**) (39, 40). Crystallographic studies of disialylated Fc reveal open and closed conformations (PDB ID codes: 4Q6Y and 5GSQ) (53, 54), and its closed conformation would be unfavorable for FcγR binding. Degalactosylation had further negative impact on FcγRIIIa binding and ADCC inhibition (**Figures 2B**, **4B, C**), due to the net loss of stabilizing oligosaccharides/protein interactions as revealed by elevated B-factor of the nongalactosylated Fc glycoform (52).

Naturally occurring galactosylated, nonfucosylated IgG in serum may be involved in immune homeostasis. Galactosylation and nonfucosylation of IgG enhance FcγRIIIa binding by two orders of magnitude (**Figure 2B**) (23, 24, 45, 46, 55), which explains why the (G2)_2_ glycoform of serum IgG bound to FcγRIIIa is not displaced by autoantibody–antigen complexes (**Figures 2B**, **5**). In the ADCC reporter bioassay, ADCC was inhibited with the (G2)_2_ glycoform of IgG at as low as 0.1 mg/ml (~0.6 μM) *in vitro* (**Figure 4B**). As the proportion of the G2 oligosaccharide released from IgG-Fc of the IVIG preparation was ~1% (**Figure 1B**, **Supplementary Figure 1** and **Supplementary Table 1**), the serum level of IgG bearing at least one G2 oligosaccharide chain is estimated to be up to 0.2 mg/ml (~1.3 μM), which is higher than the IC_50_ of the (G2)_2_ glycoform for ADCC inhibition (**Figure 4C**) and the K_d_ for the binding of the (G2)_2_ glycoform of IgG to FcγRIIIa V158 (1.98 nM) and F158 (24.6 nM) as reported previously (56). It is likely that the equilibrium of the interaction between the (G2)_2_ glycoform of serum IgG and FcγRIIIa on immune cells shifts toward association *in vivo*. In fact, the FcγRIIIa molecules isolated from circulating NK cells were shown to preferentially bind nonfucosylated IgG1 bearing G2, monosialylated G2, G1, and bisected G1 oligosaccharides while serum IgG is largely fucosylated in the same subjects (29). The imbalance of the IgG glycoform distribution between serum and FcγRIIIa on NK cells indicates that circulating galactosylated, nonfucosylated IgG glycoforms represents the tip of the iceberg. Thus, the majority of endogenous nonfucosylated IgG glycoforms are likely bound to FcγRIIIa, modulating immune cell responses in healthy conditions.

Under autoimmune and inflammatory conditions, it is conceived that circulating galactosylated, nonfucosylated IgG glycoforms decrease due to the binding to FcγRIIIa on expanding immune cells. In rheumatoid arthritis (RA), elevated hypogalactosylated IgG levels associate with disease activity (57, 58), and during pregnancy its galactosylation level can return to normal with disease symptoms being improved (58). The involvement of hypogalactosylation of serum IgG in the pathophysiology of RA remains uncertain probably because in early studies the impact of core fucosylation was not appreciated or quantitated (17). Importantly, the fucosylation level of serum IgG in RA was recently found to be elevated as compared with healthy control (58, 59), indicating a decrease of galactosylated and/or nonfucosylated IgG in serum. It should be noted that due to the asymmetry of the Fc–FcγRIIIa interaction nonfucosylation of one heavy chain is sufficient for tight binding (45, 46). Therefore, IgG bound to FcγRIIIa on immune cells may bear a pair of fucosylated and nonfucosylated oligosaccharides in the Fc portion, which may explain why a decrease of not only nonfucosylated but fucosylated oligosaccharides is observed in oligosaccharide profiles of serum IgG in RA (48). It has been reported in Guillain-Barré syndrome that the responses to IVIG therapy correlate with IgG glycosylation profiles where patients who failed to respond to IVIG were characterized by hypogalactosylation of serum IgG before and after the treatment (60). Thus, a better understanding of the relationship between glycosylation changes of IgG and disease activity will be helpful in the treatment and management of certain autoimmune diseases with IVIG and its (G2)_2_ glycoform *via* the saturation of FcγRIIIa, blocking FcγRIIIa-mediated ADCC (**Figure 5**).

To conclude, elucidation of the mechanism of action of IVIG is essential to establish its clinical indication, as over 200 metric tons of IVIG per year are consumed worldwide for treatment of autoimmune and inflammatory diseases including off-label purposes (14, 61). Considering the prioritized use of IVIG for primary immunodeficiency, the Fc fragments should suffice for immunomodulatory therapy, which suggests clinical application of glycoengineered recombinant Fc proteins as an alternative to plasma-derived IVIG. Various recombinant Fc multimers have been designed to block effector molecules including FcγRs, C1q and neonatal Fc receptor (FcRn), and some Fc multimers including GL-2045 and M230 have been under clinical evaluation (62, 63). Recombinant Fc multimers are shown to block multiple effector molecules while glycoengineered Fc monomers may not be useful to target C1q or FcRn due to low affinity to C1q (Ka = 5 x 10^4^ M^-1^) (64) and lack of the impact of Fc glycosylation on FcRn binding (40). Although recombinant Fc multimers are promising therapeutics, their broad immunomodulatory effects and unnatural antibody formats might be associated with potential risks during the long-term use in autoimmune diseases. On the other hand, galactosylated, nonfucosylated IgG glycoforms bearing human-type oligosaccharides are naturally occurring and likely devoid of immunogenicity *in vivo*. Further studies are needed to evaluate the efficacy of the (G2)_2_ glycoform of IVIG and recombinant Fc in a range of autoimmune diseases and severe infections including coronavirus disease 2019 (Covid-19) (65, 66). The disease severities of certain viral infections including SARS-CoV-2 and dengue viruses have been reported to associate with elevated levels of nonfucosylated IgG against the pathogens (67–70); therefore, the (G2)_2_ glycoform of IVIG and Fc are promising immunomodulatory agents for attenuation of antibody-dependent enhancement of infection *via* competition with antiviral nonfucosylated IgG.

## Data Availability Statement

The original contributions presented in the study are included in the article/**Supplementary Material**. Further inquiries can be directed to the corresponding author.

## Ethics Statement

The animal study was reviewed and approved by The Animal Care and Use Committees of Yamaguchi Ube Medical Center and Unitech Co., Ltd.

## Author Contributions

YM and YM-K conceived the study, designed and performed experiments, and wrote the manuscript. RS performed the glycan analysis. RJ and PR analyzed the results and cowrote the manuscript. All authors approved the manuscript.

## Conflict of Interest

The authors declare that the research was conducted in the absence of any commercial or financial relationships that could be construed as a potential conflict of interest.

## Publisher’s Note

All claims expressed in this article are solely those of the authors and do not necessarily represent those of their affiliated organizations, or those of the publisher, the editors and the reviewers. Any product that may be evaluated in this article, or claim that may be made by its manufacturer, is not guaranteed or endorsed by the publisher.

## Abbreviations

2-AB, 2-aminobenzamide; ADCC, antibody-dependent cellular cytotoxicity; CAIA, collagen antibody-induced arthritis; CRP, C-reactive protein; ENGase, endoglycosidase; FcγR, receptor for Fc portion of IgG; GlcNAc, N-acetylglucosamine; HILIC, hydrophilic interaction liquid chromatography; ITP, immune thrombocytopenia; IVIG, intravenous immunoglobulin; SGP, sialylglycopeptide; UPLC, ultraperformance liquid chromatography.
