# Erratum: Enhanced Immunomodulatory Effect of Intravenous Immunoglobulin by Fc Galactosylation and Nonfucosylation

**DOI:** 10.3389/fimmu.2022.902018

**Published:** 2022-04-01

**Authors:** Frontiers Production Office

**Affiliations:** Frontiers Media SA, Lausanne, Switzerland

**Keywords:** glycoengineering, antibody-dependent cellular cytotoxicity, intravenous immunoglobulin, autoimmune disease, natural killer cell, Fcγ receptor, oligosaccharide

Due to a production error, the measuring unit “ml” was incorrectly changed to “μl” during the production process.

A correction has been made to the section **Methods,** subsection **Preparation of Glycan Oxazolines**:

“The glycan donors sialoglycan oxazoline (S2G2-Ox), galactosylated glycan oxazoline (G2-Ox), and nongalactosylated glycan oxazoline (G0-Ox) were prepared from sialylglycopeptide (SGP) (Tokyo Chemical Industry Co. Ltd.) in a modified version of the previously described method (32). Briefly, SGP (20 mg) dissolved in 100 μl of 50 mM phosphate (pH 6.0) was digested at 37°C for 8 h with EndoS-coupled Sepharose-4 that had been prepared by coupling EndoS to CNBr-activated Sepharose-4 (GE Healthcare) to release sialoglycan, according to the manufacturer’s instruction. For G2-Ox and G0-Ox preparation, SGP (40 mg) was digested with EndoS-coupled Sepharose-4 and neuraminidase (2 U, Roche) overnight and the supernatant containing the desialylated glycan was divided into two aliquots, with one for preparation of G2-Ox and the other for G0-Ox. For the latter, the galactosylated glycan was digested with β (1-3,4)-galactosidase (Agilent) at 37°C for 48 h. The glycan in each aliquot (~100 μl) was converted to glycan oxazoline by the addition of 2-chloro-1,3-dimethylimidazolinium chloride (23.4 mg) and triethylamine (47.2 µl) on ice for 1 h. The reaction was diluted with 4 ml of butanol:ethanol:water (4:1:1, v/v/v) and purified on cellulose column (2 ml in a Poly-Prep Chromatography Column, Bio-Rad) equilibrated with the same solution (33). After washing the column with 12 ml of the solution and 2 ml of absolute ethanol, glycan oxazoline was eluted with distilled water. The glycan-containing fractions were detected with anthrone/sulfuric acid and dried under vacuum.”

The publisher apologizes for this mistake. The original version of this article has been updated.

