# Supplementary material for: Enhanced Immunomodulatory Effect of Intravenous Immunoglobulin by Fc Galactosylation and Nonfucosylation
Source: Front Immunol. 2022 Jan 28;13:818382. doi: 10.3389/fimmu.2022.818382 (PMC8831331; doi:10.3389/fimmu.2022.818382)
Supplement: Supplementary file 1 [file DataSheet_1.pdf]

Supplementary Information

Enhanced immunomodulatory effect of intravenous immunoglobulin by Fc galactosylation and nonfucosylation

Yusuke Mimura[1,*], Yuka Mimura-Kimura[1], Radka Saldova[2,3], Pauline M Rudd[2,4], and Roy Jefferis[5]

[1] Department of Clinical Research, National Hospital Organization Yamaguchi Ube Medical Center, Ube, Japan

[2] NIBRT GlycoScience Group, National Institute for Bioprocessing Research and Training, Dublin, Ireland

[3] UCD School of Medicine, College of Health and Agricultural Science, University College Dublin, Dublin, Ireland

[4] Bioprocessing Technology Institute, Agency for Science, Technology and Research, Centros, Singapore

[5] Institute of Immunology and Immunotherapy, College of Medical and Dental Sciences, University of Birmingham, Birmingham, UK

Supplementary Figure 1. HILIC-UPLC profile of human normal IgG-Fc glycans

Supplementary Figure 2. FcγRIIa-mediated antibody-dependent cellular phagocytosis (ADCP) was not modulated by the IgG glycoforms

Supplementary Figure 3. Low-dose $(G2)_2$ glycoform of IVIG attenuated collagen antibody-induced arthritis in mice

Supplementary Table 1. Summary of human normal IgG-Fc glycans

Method

Structural characterization of IgG-Fc glycans

The oligosaccharide peaks of human normal IgG purified from a commercial IVIG (Gammagard, Shire Japan) were assigned, according to previous studies (1-3). Briefly, 2-AB-labeled oligosaccharides were digested using arrays of the following enzymes: *Arthrobacter ureafaciens* sialidase (New England Biolabs, Cat. No. P0722L), bovine testis β-galactosidase (Prozyme, Cat. No. GKX-5013), bovine kidney α-fucosidase (New England Biolabs, Cat. No. P0748L), β-*N*-acetylglucosaminidase cloned from *Streptococcus pneumoniae*, expressed in *Escherichia coli* (Prozyme, Cat. No. GK80050). After incubation at 37 °C for 16 h, the enzymes were removed by filtration using 10 kDa spin filters (Pall Corp.). The undigested and digested glycans were analyzed by HILIC-UPLC, and the elution times of glycans are expressed in glucose units (GU) by reference to a dextran ladder. Each individual glycan has a GU value that is directly related to the number and linkage of its constituent monosaccharides. The use of arrays of exo-glycosidases in combination with undigested glycan profiling enables the identification

of individual monosaccharides and linkages through enzyme specificity.

Antibody-dependent cellular phagocytosis (ADCP) reporter bioassay

ADCP reporter bioassay mediated by FcγRIIa H131 was performed, according to the manufacturer's instruction (Promega, cat. no. G9901). Briefly, CD20-expressing Raji cells grown in RPMI1640 cell culture medium supplemented with 10% heat-inactivated fetal bovine serum (Gibco), 2 mM glutamine, 100 μg/ml penicillin and 100 U/ml streptomycin (10% RPMI) were plated after washing once with PBS and resuspended in RPMI1640 medium containing 4% fetal bovine serum, ultra-low IgG (Life Technologies) at 12,500 cells/25 μl/well in white opaque tissue culture plates (BD Falcon 353296), followed by the addition of 25 μl of rituximab (anti-CD20 IgG) that was 2.5-fold serially diluted from the starting concentration of 3 μg/ml with the same medium. Increasing concentrations of native IgG (0, 1, 2, 3, and 10 mg/ml) or an individual glycoform of IgG (1 mg/ml) was added to each well (7.5 μl/well). Genetically engineered Jurkat T cells stably expressing human FcγRIIa H131 and NFAT-luciferase reporter in 10% RPMI were added at 75,000 cells/17.5 μl/well to rituximab-opsonized Raji cells at 37 °C, 5% humidified CO_2 for 6 h. BioGlo luciferase assay reagent was added (75 μl/well), and chemiluminescence was measured with a luminometer (Fluoroskan Ascent FL, Thermo Fisher Scientific).

Collagen antibody-induced arthritis (CAIA)

DBA/1 J mice (7 weeks old, female) were purchased from Japan SLC, Inc. (Shizuoka, Japan) and housed in the specific pathogen-free animal research facility of Unitech Co., Ltd. (Chiba, Japan) for experimentation. The mice at 8 weeks of age were administered

from the tail vein with 1.5 mg of anti-collagen antibody cocktail consisting of five monoclonal antibodies (A2-10, F10-21, D8-6, D1-2G, and D2-112, Chondrex Inc.) on day 0. On day 3, $(G2)_2$, $(S2G2)_2$, $(S2G2F)_2$, or native IgG at 6 mg/ml PBS (low dose, 0.1 g/kg), native IgG at 60 mg/ml PBS (high dose, 1 g/kg) or PBS was administered from the tail vein 1 h before intraperitoneal injection of lipopolysaccharide (12.5 μg) (n=3/group). Development of arthritis was monitored up to day 8 and qualitatively assessed by investigators who were blinded to the treatment groups as below: 0 (normal), 1 (redness and swelling limited to digits), 2 (moderate redness and swelling of ankle or wrist), 3 (severe redness and swelling of the entire paw), 4 (maximally inflamed limb involving multiple joints). The sum of the arthritic scores of all four paws were compared between the groups. At termination of the experiment on day 8, mice were euthanized under deep anesthesia with isoflurane. Blood was taken from the heart, and the sera were kept at -80 °C until use. The serum levels of interleukin (IL)-1β, IL-6, tumor necrosis factor (TNF)-α were quantitated by Bio-Plex multiplex assay (Bio-Rad) and that of C-reactive protein (CRP) with Quantikine Mouse C-Reactive Protein ELISA kit (R&D Systems). The hind paws were fixed in 10% (w/v) neutral buffered formalin, decalcified in 5% formic acid for 7 days, and embedded in paraffin. Frontal sections of the ankle joints of the hind paws were stained with hematoxylin/eosin. The animal experiment was designed in compliance with the guideline of Science Council of Japan, approved by The Animal Care and Use Committees of Yamaguchi Ube Medical Center (Approval No. 2020-1) and Unitech Co., Ltd. (Approval No. AGR YGU-200520A-80). One-way ANOVA with Tukey's multiple-comparison test was used to analyze the differences in the serum levels of the inflammatory marker (SPSS, ver. 25). $p < 0.05$ was considered statistically significant.

References

1. Pucic M, Knezevic A, Vidic J, Adamczyk B, Novokmet M, Polasek O, et al. High throughput isolation and glycosylation analysis of IgG-variability and heritability of the IgG glycome in three isolated human populations. *Mol Cell Proteomics* (2011) 10:M111 010090. doi:10.1074/mcp.M111.010090

2. Doherty M, McManus CA, Duke R and Rudd PM. High-throughput quantitative N-glycan analysis of glycoproteins. *Methods Mol Biol* (2012) 899:293-313. doi:10.1007/978-1-61779-921-1_19

3. Hilliard M, Alley WR, Jr., McManus CA, Yu YQ, Hallinan S, Gebler J, et al. Glycan characterization of the NIST RM monoclonal antibody using a total analytical solution: From sample preparation to data analysis. *MAbs* (2017) 9:1349-59. doi:10.1080/19420862.2017.1377381

4. Washburn N, Schwab I, Ortiz D, Bhatnagar N, Lansing JC, Medeiros A, et al. Controlled tetra-Fc sialylation of IVIg results in a drug candidate with consistent enhanced anti-inflammatory activity. *Proc Natl Acad Sci U S A* (2015) 112:E1297-306. doi:10.1073/pnas.1422481112

Supplementary Figure 1

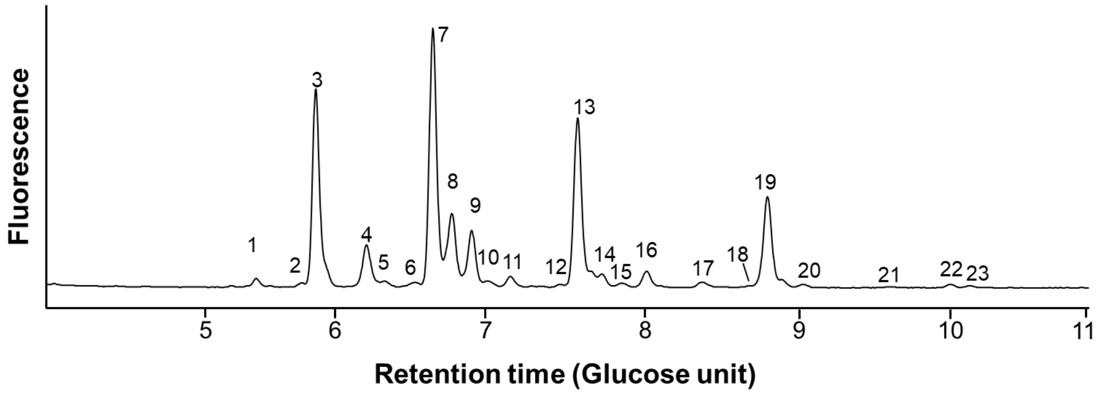

Supplementary Figure 1. HILIC-UPLC profile of human normal IgG-Fc glycans**.** The glycan profile is adapted from that of native IgG-Fc in Figure 1B. The elution times of glycans are expressed in glucose units (GU) by reference to a dextran ladder. The assigned IgG-Fc glycans are listed in Supplementary Table 1.

Supplementary Figure 2

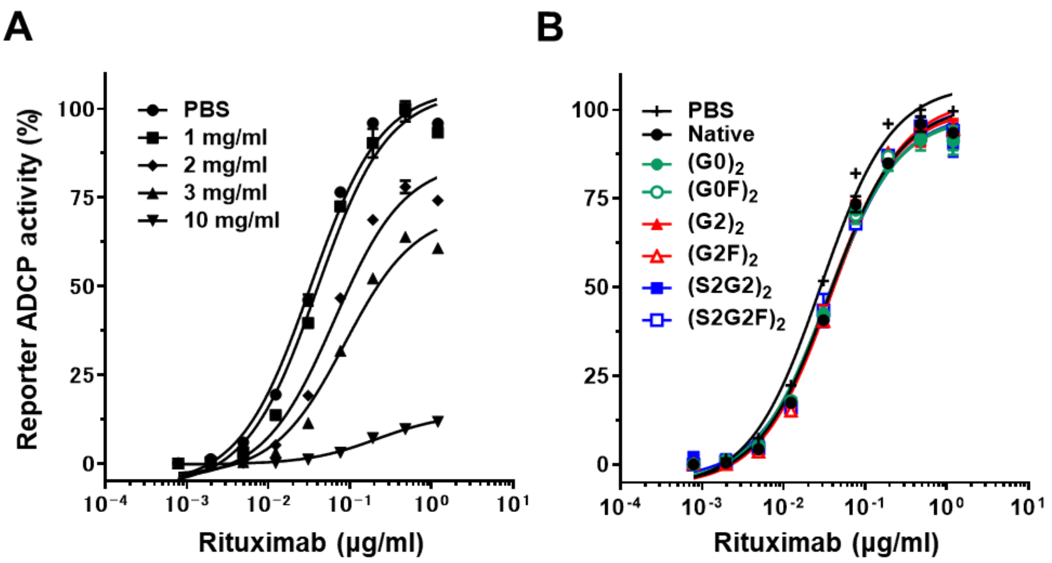

Supplementary Figure 2. FcγRIIa-mediated antibody-dependent cellular phagocytosis (ADCP) was not modulated by the IgG glycoforms. ADCP reporter bioassay was performed in the presence of normal IgG. (A) Dose-dependent inhibition of ADCP by normal IgG at 0 – 10 mg/ml. (B) No significant difference in ADCP inhibition by the IgG glycoforms at 1 mg/ml.

Supplementary Figure 3

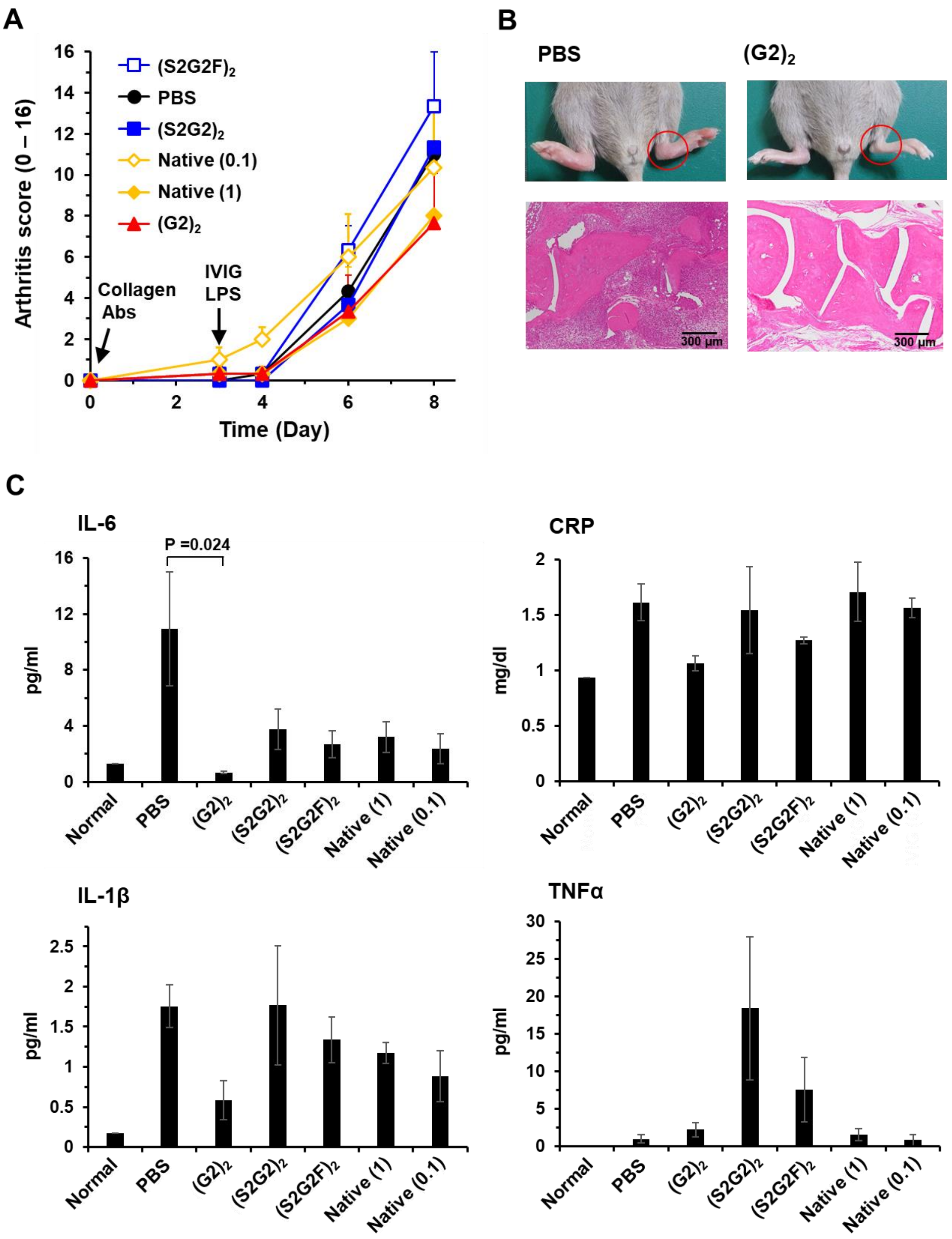

Supplementary Figure 3. Low-dose $(G2)_2$ glycoform of IVIG attenuated collagen antibody-induced arthritis in mice. (A) Time course of the arthritis scores of arthritic mice treated with IVIG glycoforms. Error bars, mean + S.E. (n = 3). (B) Histology of the hind paws of arthritic mice treated with either PBS or the $(G2)_2$ glycoform of IVIG (H&E staining, scale bars 300 μm). (C) Serum levels of inflammatory markers IL-6, C-reactive protein (CRP), IL-1β, and TNFα on day 8. Low-dose (0.1 g/kg) of the IgG glycoforms [$(G2)_2$, $(S2G2)_2$, $(S2G2F)_2$, native] and high-dose (1 g/kg) of native IgG as positive control were administered to DBA/1J mice 3 days after arthritis was induced with the collagen antibody cocktail. Five days after the administration of IVIG (day 8), the group receiving the $(G2)_2$ glycoform exhibited the lowest arthritis score among the groups (A). The histology of the hind paws revealed cartilage and bone erosion/destruction and massive inflammatory cell infiltration in the PBS-treated mice whereas markedly lower inflammatory states were observed in the $(G2)_2$-treated mice on day 8 (B). The serum levels of IL-6 in mice receiving the $(G2)_2$ were significantly lower than those receiving PBS whereas neither the $(S2G2)_2$ nor $(S2G2F)_2$ glycoform was protective in contrast to the previous report (4) (C). Error bars, mean ± S.E. (n = 3).

Supplementary Table 1

Supplementary Table 1. Summary of human normal IgG-Fc glycans.

Peak no.	Peak composition[a]	Glycan name	Structure	GU[b]	% Area
1	A2	G0	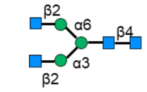	5.40	0.82
2	A2B	G0B	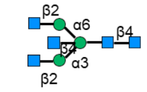	5.76	0.34
3	F(6)A2	G0F	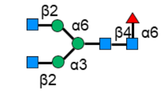	5.87	18.19
4	F(6)A2B	G0FB	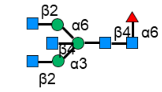	6.24	3.98
	A2[6]G1	G1[6]	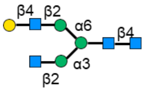	6.24	0.74
5	A2[3]G1	G1[3]	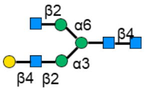	6.36	0.63
6	A2BG1	G1B	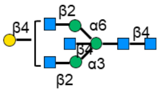	6.57	0.65
7	F(6)A2[6]G1	G1F[6]	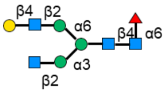	6.69	23.85
8	F(6)A2[3]G1	G1F[3]	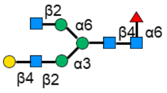	6.81	8.15
9	F(6)A2[6]BG1	G1FB[6]	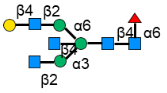	6.94	5.92
10	F(6)A2[3]BG1	G1FB[3]	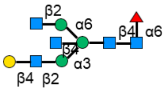	7.04	0.74
11	A2G2	G2	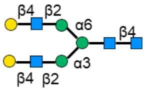	7.18	1.22
12	A2BG2	G2B	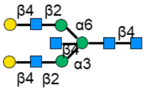	7.48	0.27

13	F(6)A2G2	G2F	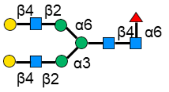	7.59	17.22
14	F(6)A2BG2	G2FB	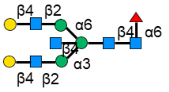	7.73	1.90
15	F(6)A2[6]G1S1	S1G1F[6]	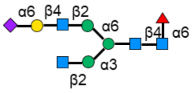	7.86	0.56
16	F(6)A2[3]G1S1	S1G1F[3]	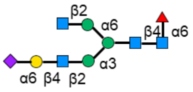	8.01	1.40
	F(6)A2[6]BG1S1	S1G1FB[6]	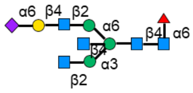	8.01	0.56
17	A2G2S1	S1G2	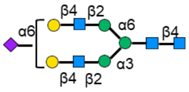	8.35	0.59
	F(6)A2[3]BG1S1	S1G1FB[3]	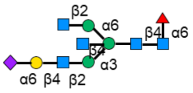	8.35	0.21
18	A2BG2S1	S1G2B	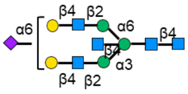	8.56	0.20
19	F(6)A2G2S1	S1G2F	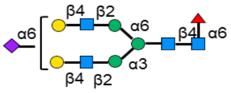	8.77	10.58
20	F(6)A2BG2S1	S1G2FB	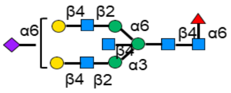	9.00	0.40
21	A2G2S2	S2G2	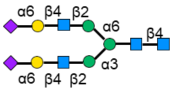	9.59	0.20
22	F(6)A2G2S2	S2G2F	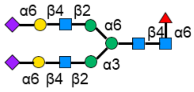	10.01	0.39
23	F(6)A2BG2S2	S2G2FB	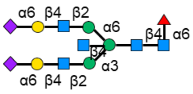	10.15	0.30

[a] Nomenclature: F: fucose; G: galactose; B: bisecting GlcNAc; S: sialic acid; A2: biantennary core heptasaccharide (G0). Numbers with parentheses indicate the preceding monosaccharide's linkage and those in brackets define to which core mannose is extended, if needed to be defined. Numbers not in parentheses indicate the amount of the preceding feature.

[b] GU: glucose unit
